# Supplementary material for: Molecular-Clinical Correlation in Pediatric Medulloblastoma: A Cohort Series Study of 52 Cases in Taiwan
Source: Cancers (Basel). 2020 Mar 11;12(3):653. doi: 10.3390/cancers12030653 (PMC7139704; doi:10.3390/cancers12030653)
Supplement: Supplementary file 1 [file cancers-12-00653-s001.zip › cancers-720756-suppl-XML/cancers-720756-suppl-Figures and Table S1.pdf]

Supplementary Materials: Molecular-Clinical Correlation in Pediatric Medulloblastoma: A Cohort Series Study of 52 Cases in Taiwan

Kuo-Sheng Wu, Donald Ming-Tak Ho, Shiann-Tarng Jou, Alice L. Yu, Huy Minh Tran, Muh-Lii Liang, Hsin-Hung Chen, Yi-Yen Lee, Yi-Wei Chen, Shih-Chieh Lin, Feng-Chi Chang, Min-Lan Tsai, Yen-Lin Liu, Hsin-Lun Lee, Kevin Li-Chun Hsieh, Wen-Chang Huang, Shian-Ying Sung, Che-Chang Chang, Chun Austin Changou, Kung-Hao Liang, Tsung-Han Hsieh, Yun-Ru Liu, Meng-En Chao, Wan Chen [1], Shing-Shung Chu, Er-Chieh Cho and Tai-Tong Wong

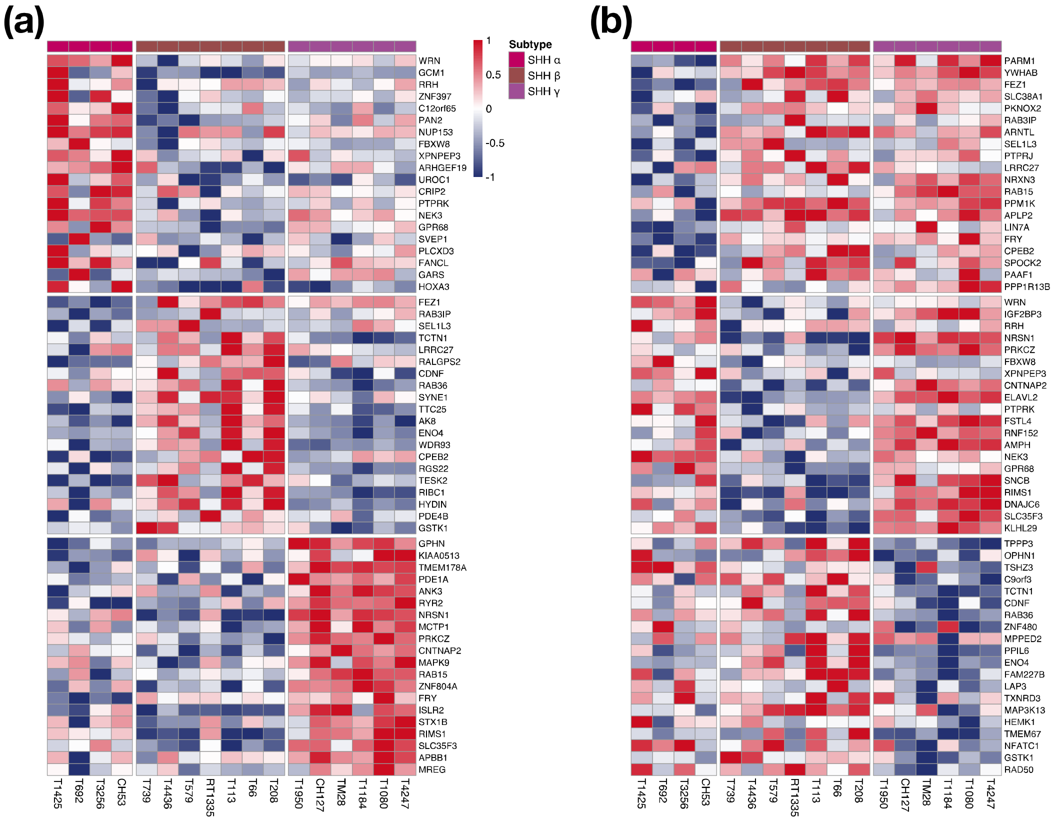

Figure S1. Heatmap of top (**a**) and bottom (**b**) 20 genes in the three SHH subtype.

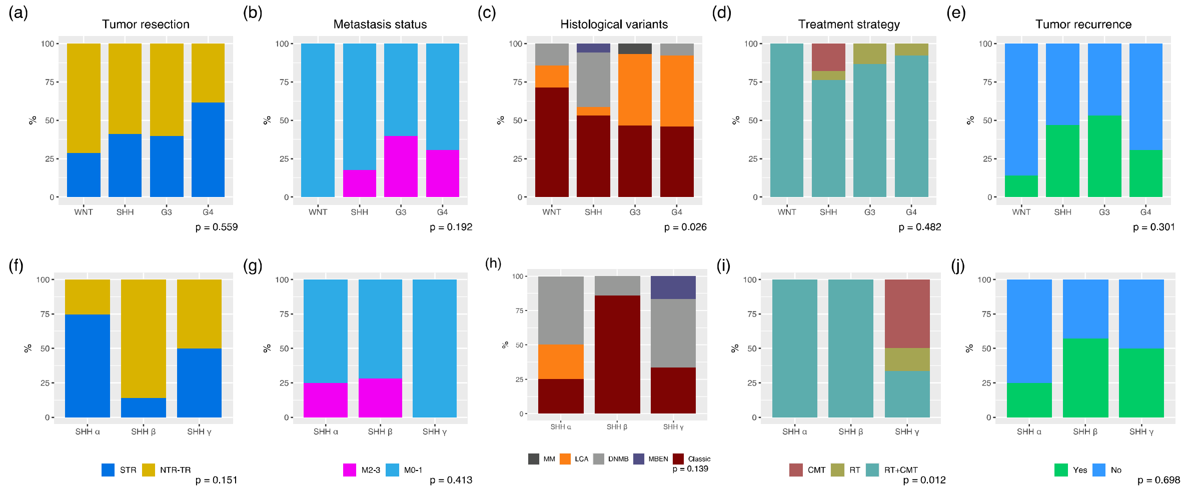

Figure S2. The subgroup distribution of clinical parameters in the four core MB subgroups: (**a**) Extent of tumor resection (Fisher's exact test) split into yellow (near-total resection/total resection; NTR-TR)

and blue (subtotal resection; STR). (**b**) Metastasis at diagnosis (Fisher's exact test) split into blue (M0-1) and red (M2-3). (**c**) Histological subtypes (Fisher's exact test) split into brown (Classic MB), orange (LCA MB), grey (DNMB), navy blue (MBEN), and dark grey (MMMB). (**d**) Treatment strategy (Fisher's exact test) split into brown (CMT), yellowish brown (RT), green lake (RT+CMT). (**e**) Tumor recurrence (Fisher's exact test) split into blue (No) and green (Yes). The subtype distribution of clinical parameters in the three pediatric SHH subtypes: (**f**) Extent of tumor resection (Fisher's exact test) split into yellow (NTR-TR) and blue STR. (**g**) Metastasis at diagnosis (Fisher's exact test) split into blue (M0-1) and red (M2-3). (**h**) Histological subtypes (Fisher's exact test) split into brown (Classic MB), orange (LCA MB), grey (DNMB), and navy blue (MBEN). (**i**) Treatment strategy (Fisher's exact test) split into brown (CMT), yellowish brown (RT), and green lake (RT+CMT). (**j**) Tumor recurrence (Fisher's exact test) split into blue (No) and green (Yes).

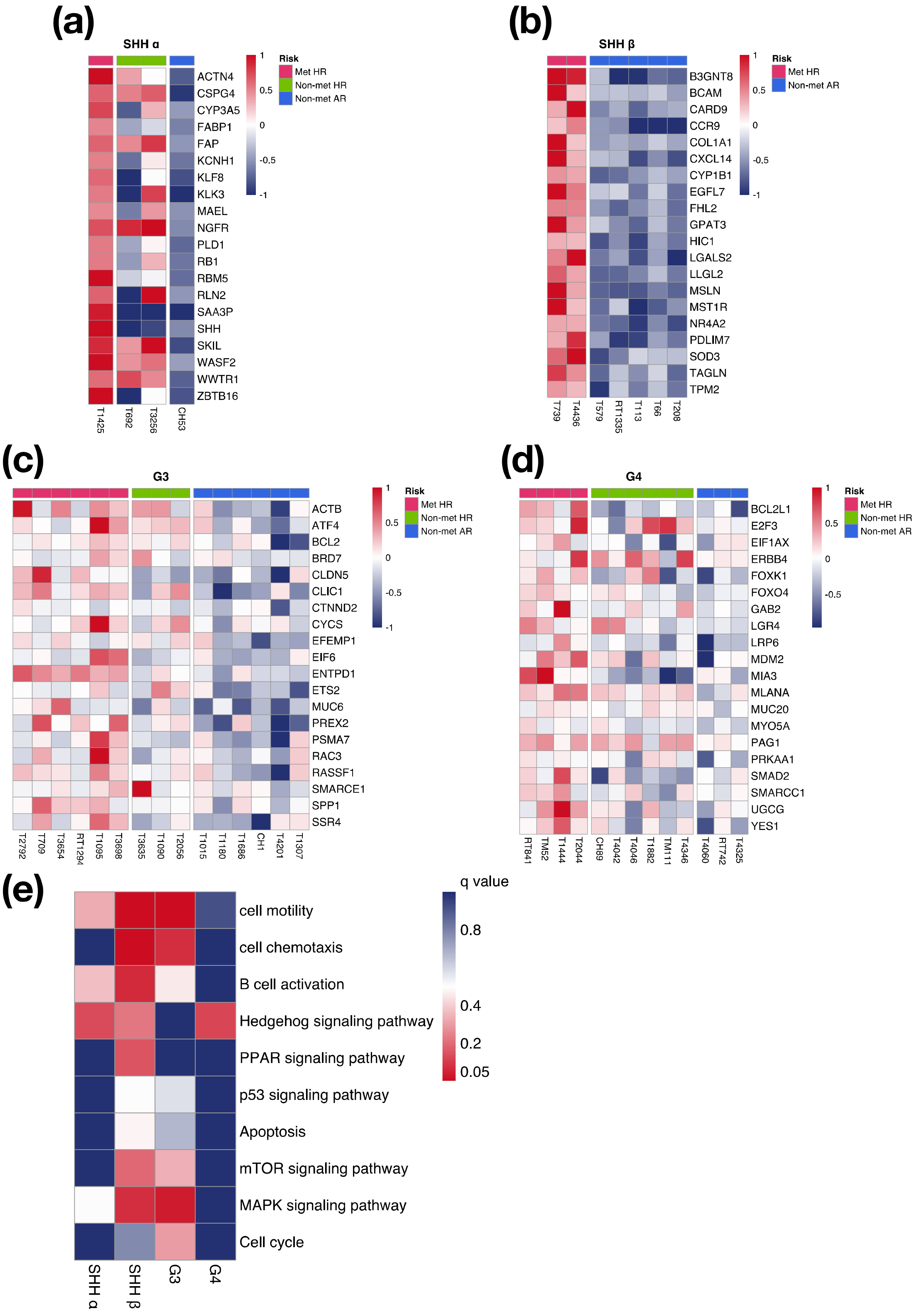

Figure S3. Top 20 highly expressed metastasis-associated genes in tumors with metastasis in SHH α (**a**), SHH β (**b**), Group 3 (**c**), and Group 4 (**d**). (**e**) Gene set enrichment analysis of pathway in tumors with metastasis in MB subgroups.

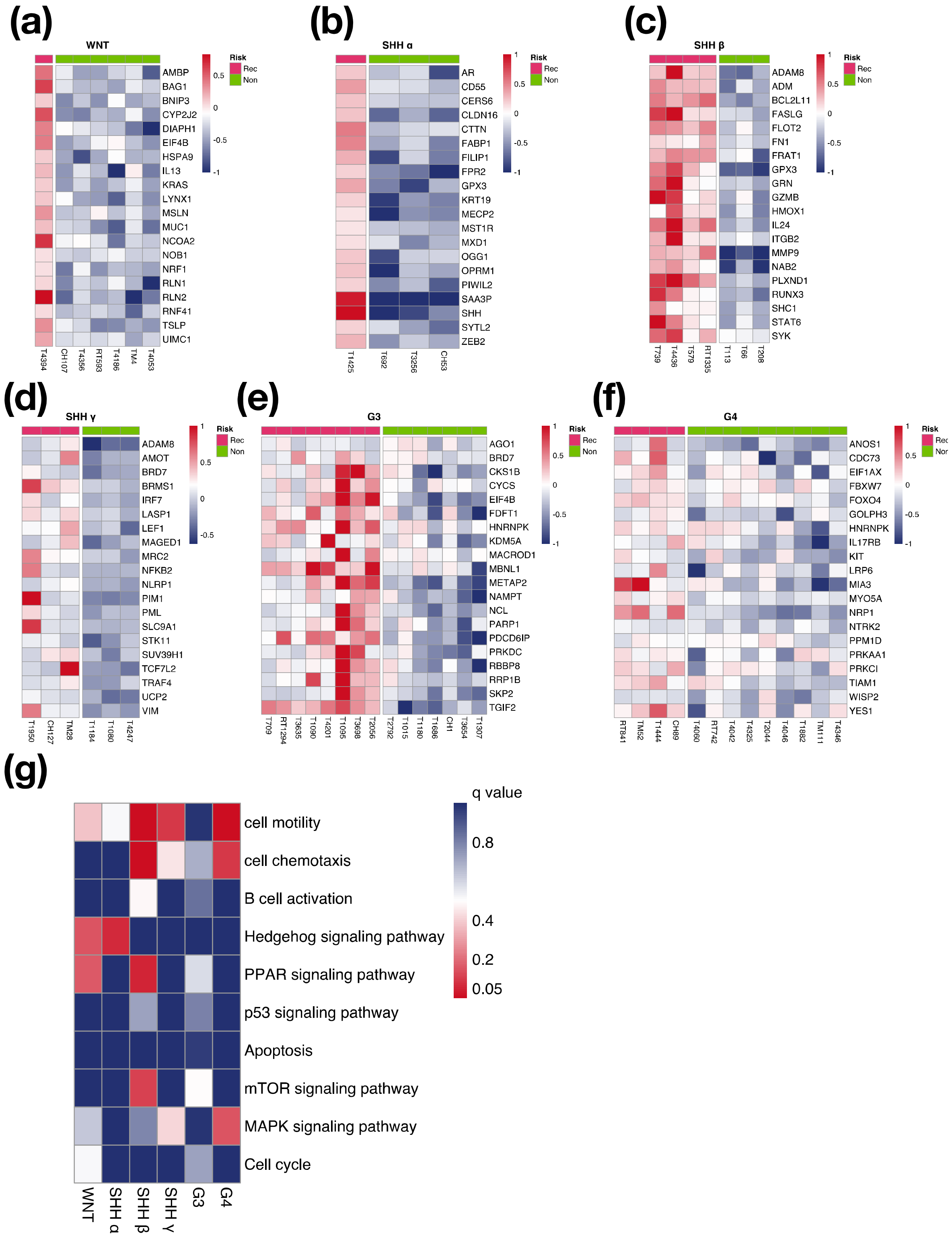

Figure S4. Top 20 highly expressed metastasis-associated genes in tumors with recurrence in WNT (**a**), SHH α (**b**), SHH β (**c**), SHH γ (**d**), Group 3 (**e**), and Group 4 (**f**). (**g**) Gene set enrichment analysis of signature pathway in tumors with recurrence in MB subgroups.

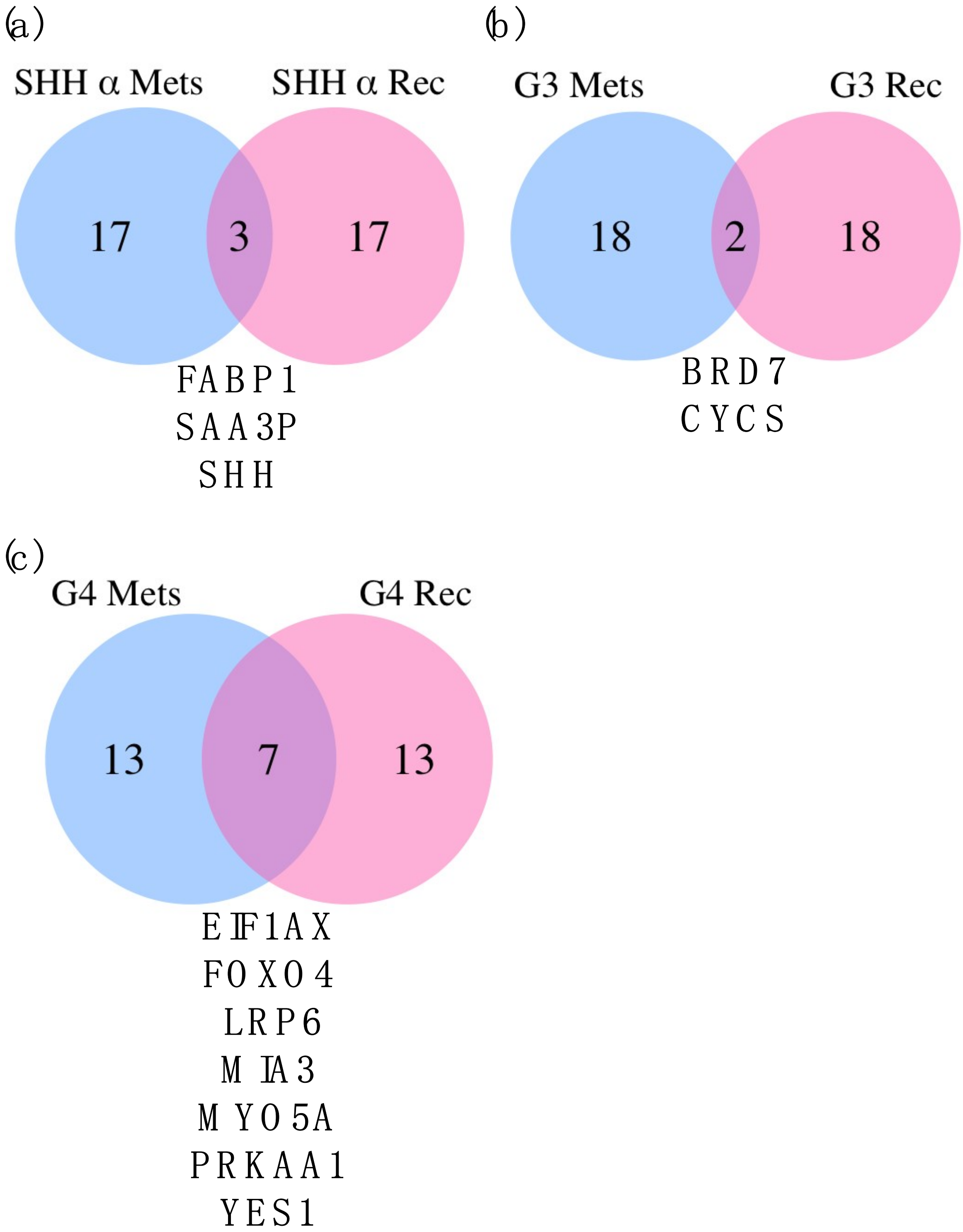

Figure S5. The overlapping genes in tumors with metastasis and recurrence in SHH *α* (**a**), Group 3 (**b**), and Group 4 (**c**).

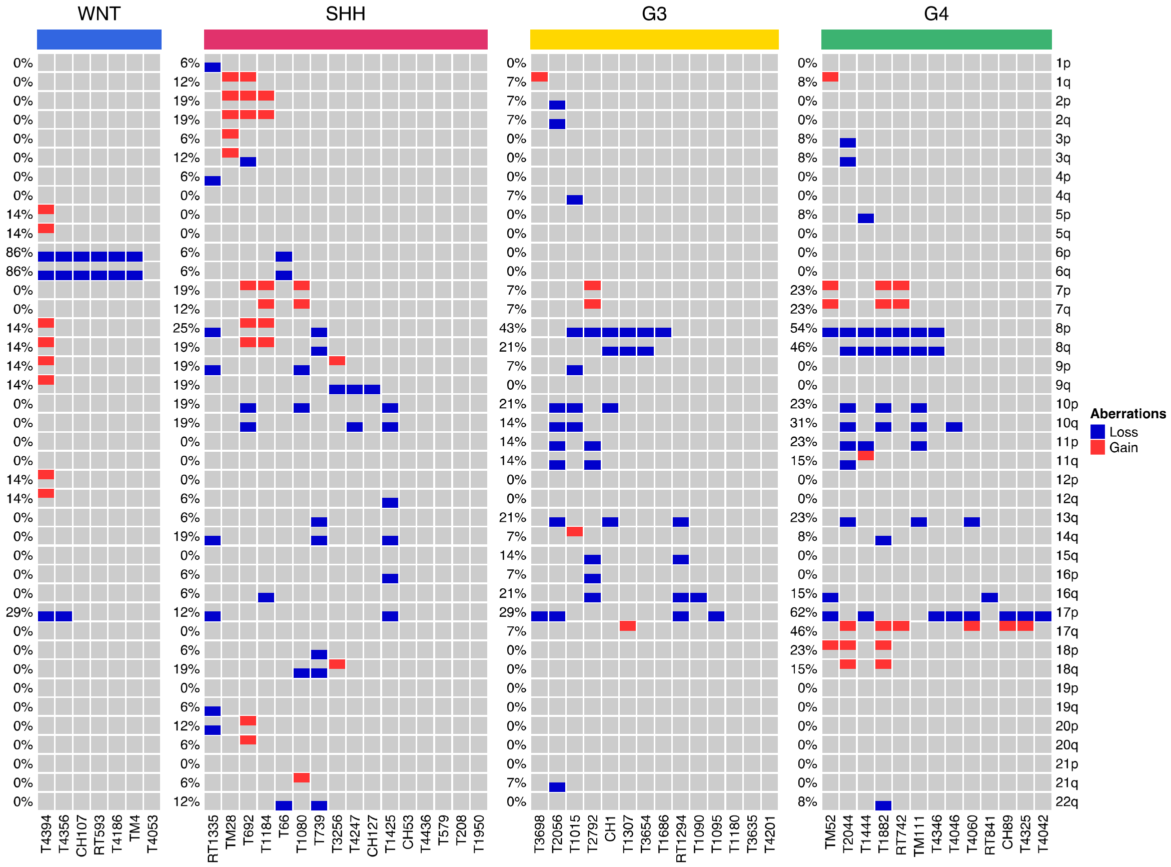

Figure S6. Subgroup distribution of chromosome aberrations in 50 MBs with DNA methylation analysis split into blue (loss) and red (gain).

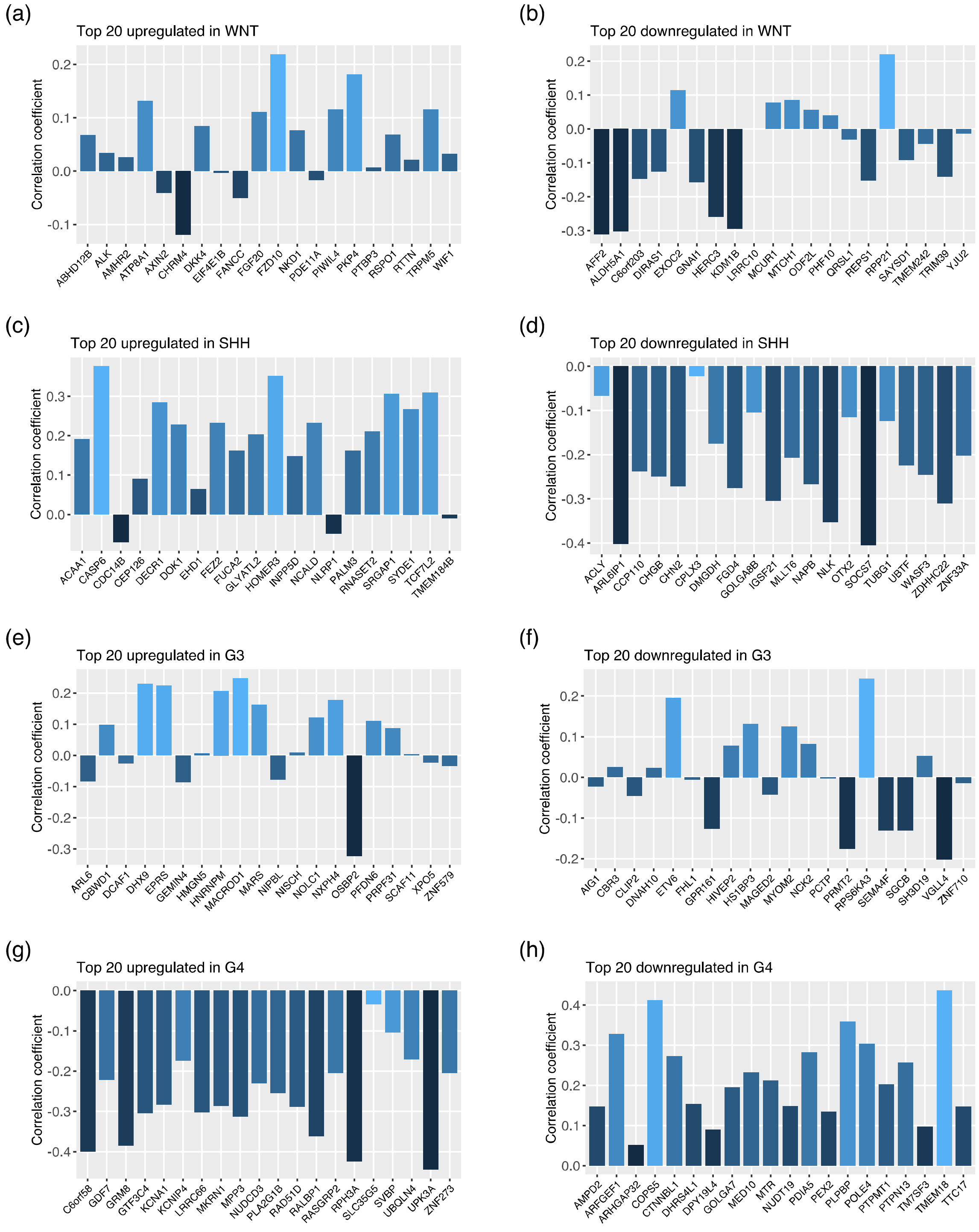

Figure S7. The correlation coefficient of top 20 upregulated and downregulated genes in WNT (**a, b**), SHH (**c, d**), Group 3 (**e, f**), and 4 (**g, h**) MBs and tumor mutation burden.

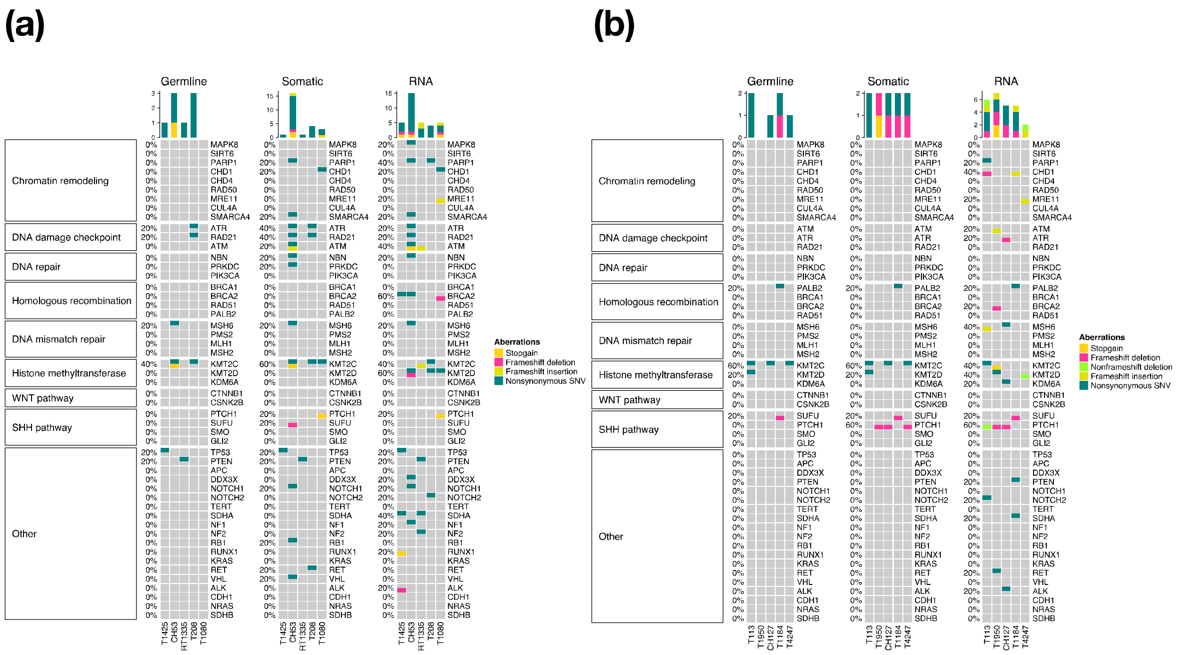

Figure S8. Somatic and germline variant calling obtained from RNA-Seq and WES data in 10 of the 17 SHH MBs in the cohort series of 52 childhood MBs in Taiwan. (**a**) Three of the five cases with relevant clinical findings showed germline mutations of *TP53*, *MSH6*, and *PTEN*. (**b**) One of the five cases without relevant clinical findings showed *SUFU* germline mutation.

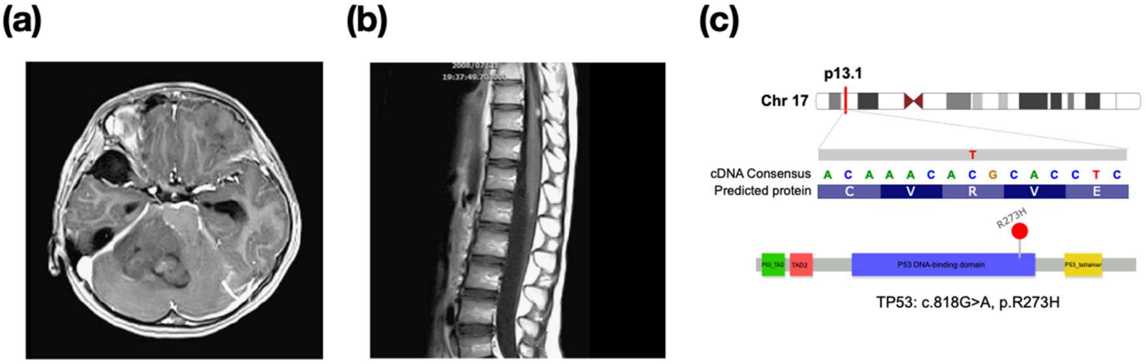

Figure S9. A case of M2 SHH α MB, large cell anaplastic histologic variant (LCA), with germline *TP53* mutation (Case 1, Table 4). (**a**, **b**) Cerebellar-pontine angle tumor and spinal metastasis (**c**) *TP53* mutation loci.

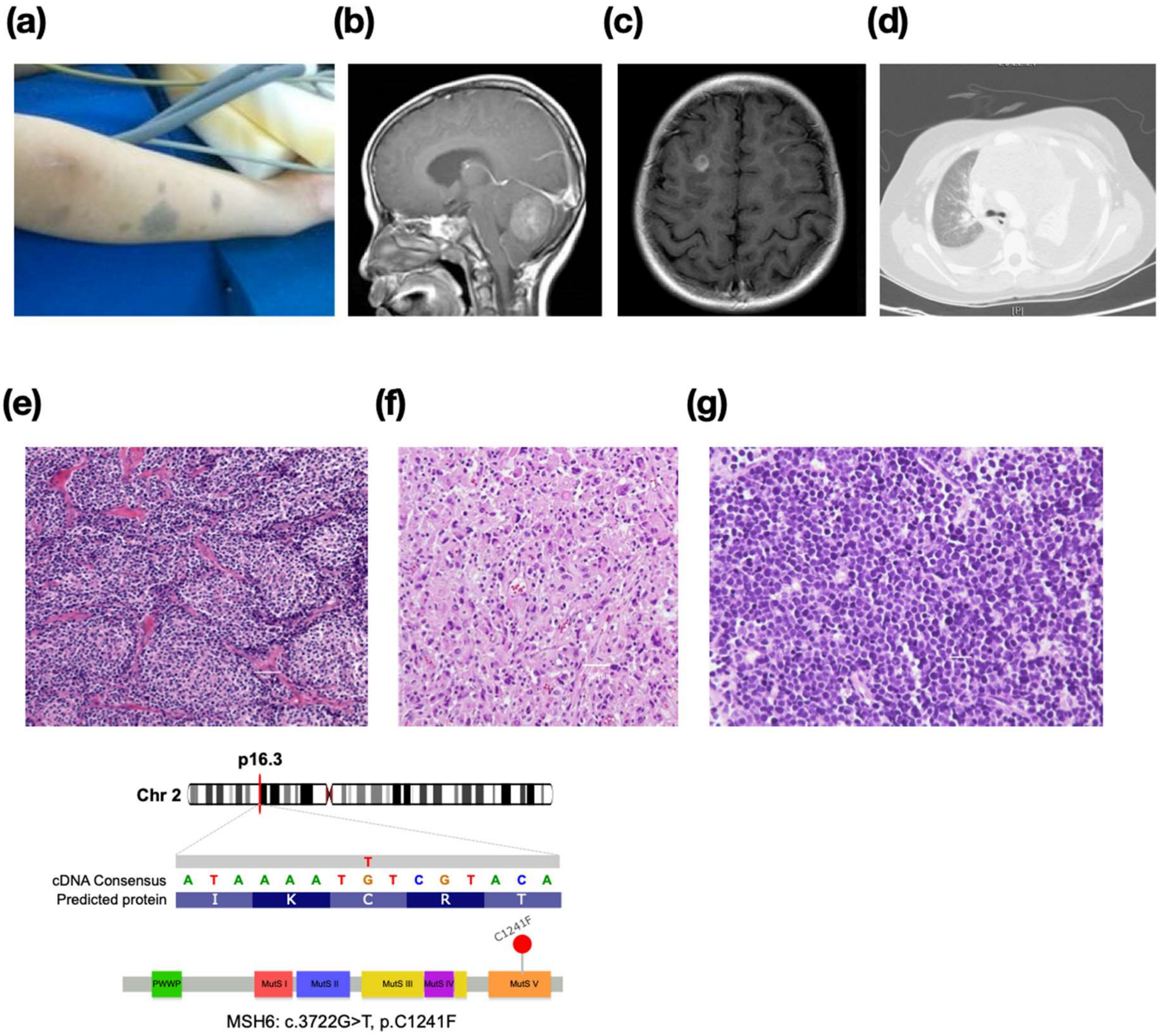

Figure S10. A case of M0–1 SHH α MB, desmoplastic/nodular (DN) histologic variant, with biallelic *MSH6* germline mutation and three heterochronous malignancies: MB, anaplastic astrocytoma, and T-lymphoblastic leukemia. (**a**) Blue nevi in right forearm. (**b**) Cerebellar vermis MB. (**c**) Right frontal anaplastic astrocytoma. (**d**) Left-side lung T-lymphoblastic leukemia with pleural effusion. (**e**) DNMB pathology. (**f**) Anaplastic astrocytoma pathology. (**g**) Lymph node with T-cell leukemia. (**h**) Biallelic *MSH6* germline mutation loci. Scale bar: (**e**) 40 µm, (**f**) 20 µm, (**g**) 20 µm.

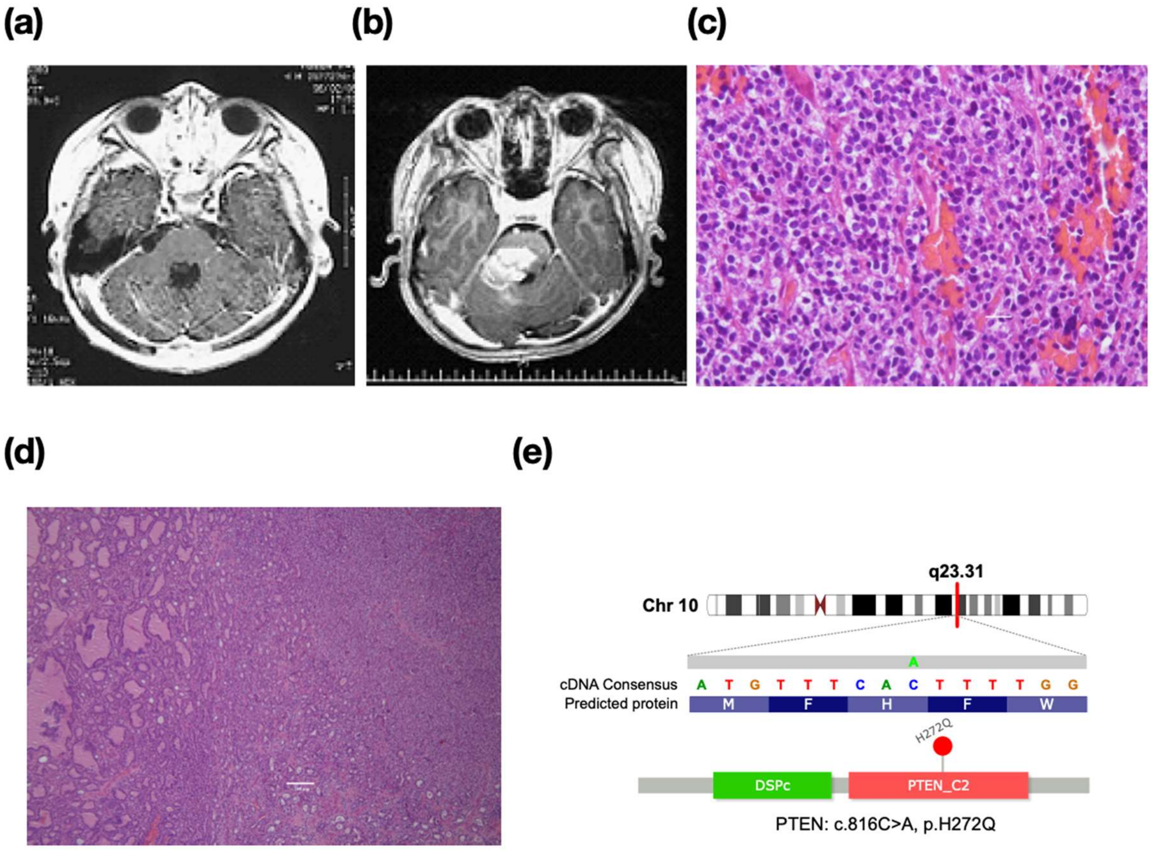

Figure S11. A case of M0–1 SHH β MB, classic histologic variant, *PTEN* germline mutation with. synchronous diagnosis of recurrent MB and multiple adenomatous hyperplasias and nodular goiters (Case 3, Table 4). **(a)** MRI of brain after gross total resection. **(b)** MRI of local recurrence at 16 years after postresection radiotherapy and chemotherapy. **(c)** MB pathology at recurrence. **(d)** Pathology of multiple adenomatous hyperplasia with nodular goiter. **(e)** *PTEN* germline mutation loci. Scale bar: (**c**) 20 μm, (**d**) 200 μm.

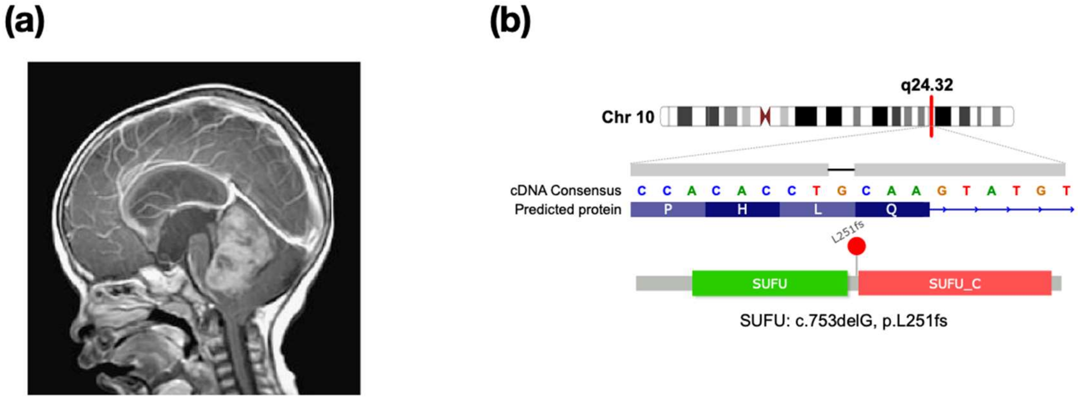

Figure S12. A case of M0-1 SHH γ MB, desmoplastic/nodular (DN) histologic variant, with *SUFU* germline with no record of relevant clinical findings. (Case 6, Table 4). (**a**) MRI of brain at diagnosis. (**b**) *SUFU* germline mutation loci.

Table S1. The correlation coefficient of top 20 upregulated and downregulated genes in the four core subgroups of MBs and tumor mutation burden.

Subgroup	Expression	Correlation (Range)	*p* Value (Range)
WNT	up	−0.119~0.219	0.119~0.980
WNT	down	−0.310~0.220	0.025~0.999
SHH	up	−0.069~0.376	0.006~0.944
SHH	down	−0.405~−0.022	0.003~0.877
G3	up	−0.322~0.247	0.020~0.982
G3	down	−0.202~0.243	0.083~0.987
G4	up	−0.444~−0.035	0.001~0.806
G4	down	0.051~0.437	0.001~0.721

© 2020 by the authors. Licensee MDPI, Basel, Switzerland. This article is an open access article distributed under the terms and conditions of the Creative Commons Attribution (CC BY) license (http://creativecommons.org/licenses/by/4.0/).
